# A Quick Classifying Method for Tracking and Erosion Resistance of HTV Silicone Rubber Material via Laser-Induced Breakdown Spectroscopy

**DOI:** 10.3390/s19051087

**Published:** 2019-03-03

**Authors:** Ping Chen, Xilin Wang, Xun Li, Qishen Lyu, Naixiao Wang, Zhidong Jia

**Affiliations:** 1 Engineering Laboratory of Power Equipment Reliability in Complicated Coastal Environment, Graduate School at Shenzhen, Tsinghua University, Shenzhen 518055, China; cp17@mails.tsinghua.edu.cn (P.C.); wnx17@mails.tsinghua.edu.cn (N.W.); jiazd@sz.tsinghua.edu.cn (Z.J.); 2 Shenzhen Power Supply Co. Ltd., Shenzhen 518038, China; xunli@whu.edu.cn (X.L.); lvqishen@126.com (Q.L.)

**Keywords:** silicone rubber material, tracking and erosion resistance, laser-induced breakdown spectroscopy, neural network

## Abstract

Silicone rubber material is widely used in high-voltage external insulation systems due to its excellent hydrophobicity and hydrophobicity transfer performance. However, silicone rubber is a polymeric material with a poor ability to resist electrical tracking and erosion; therefore, some fillers must be added to the material for performance enhancement. The inclined plane test is a standard method used for evaluating the tracking and erosion resistance by subjecting the materials to a combination of voltage stress and contaminate droplets to produce failure. This test is time-consuming and difficult to apply in field inspection. In this paper, a new and faster way to evaluate the tracking and erosion resistance performance is proposed using laser-induced breakdown spectroscopy (LIBS). The influence of filler content on the tracking and erosion resistance performance was studied, and the filler content was characterized by thermogravimetric analysis and the LIBS technique. In this paper, the tracking and erosion resistance of silicone rubber samples was correctly classified using principal component analysis (PCA) and neural network algorithms based on LIBS spectra. The conclusions of this work are of great significance to the performance characterization of silicone rubber composite materials.

## 1. Introduction

Insulators of high-voltage transmission lines usually gather large amounts of contamination on their surface after long-term operation in an outdoor environment [1]. The contamination becomes wet and forms a conductive water film on the insulator surface in wet weather, which is prone to causing flashover. Silicone rubber materials are widely used in electrical power systems for their excellent hydrophobicity and hydrophobicity transfer performance compared with ceramic materials, which is a key point in the anti-pollution flashover problem [2,3]. Silicone rubber materials are mainly used in the external insulation field, such as in high temperature vulcanized (HTV) silicone rubber insulators and room temperature vulcanized (RTV) silicone rubber coatings.

However, silicone rubber insulators also face challenges when serving in outdoor environments. Usually, dry band arcing is generated on the insulator surface due to the contamination when the hydrophobicity of insulators is temporarily lost, and then the silicone rubber material will be ablated due to thermal degradation. Fillers are widely added to silicone rubber material to improve its tracking and erosion resistance performance [4,5,6]. As an important indicator of the service lifetime of silicone rubber materials, tracking and erosion resistance performance has great significance for the operation and maintenance of transmission lines.

A conventional method used to measure tracking and erosion resistance performance is the inclined plane test method. This method simulates the thermal degradation of materials by electric arcing [7,8]. However, this method is complicated and time-consuming. It is difficult to apply in field inspection because this method is mainly used for factory inspection and only rarely for insulators in service when an entire insulator string is removed from a transmission line and destructive sampling must be carried out. Cherney et al. developed a new method to replace the inclined plane test method by simulating the thermal degradation process of materials via a continuous laser ablation process [9,10]. It is more convenient than the inclined plane test, but still requires a long test time.

The laser-induced breakdown spectroscopy (LIBS) technique is an elemental analysis technique that induces the sample to generate plasma by focusing an intense pulsed laser onto the sample surface [11,12]. LIBS has been applied in various fields because of its advantages, which include the lack of a sample preparation requirement, fast measuring speed, ability to detect almost all elements and so on. The LIBS technique has great application potential in high-voltage external insulation detection due to these advantages and the remote measurement ability [13,14]. Via LIBS, it is possible to realize on-site detection of operating insulators, which is attractive for daily operation and maintenance.

In this work, the relationship between the tracking and erosion resistance performance of silicone rubber materials and the corresponding aluminum hydroxide (ATH) and silica filler content will be studied. The measurement results of filler content via TGA and LIBS were compared. The conventional method was compared with a neural network algorithm when the material properties were studied via LIBS.

## 2. Materials and Methods

### 2.1. Materials

In this work, 27 types of HTV silicone rubber samples with different contents of ATH and silica filler were prepared for testing. Silicone rubber mainly contains siloxane, additive agent like ATH and silica fillers, and other assistance like vulcanizing agent, iron oxide and so on. The ingredient information of samples is shown in Table 1. All of the samples were prepared under the same manufacturing procedure except for the content of the two fillers mentioned above. The unit of content is relative mass, while the resin content of each sample is normalized to 1.

### 2.2. Inclined Plane Test

**Figure 1:**
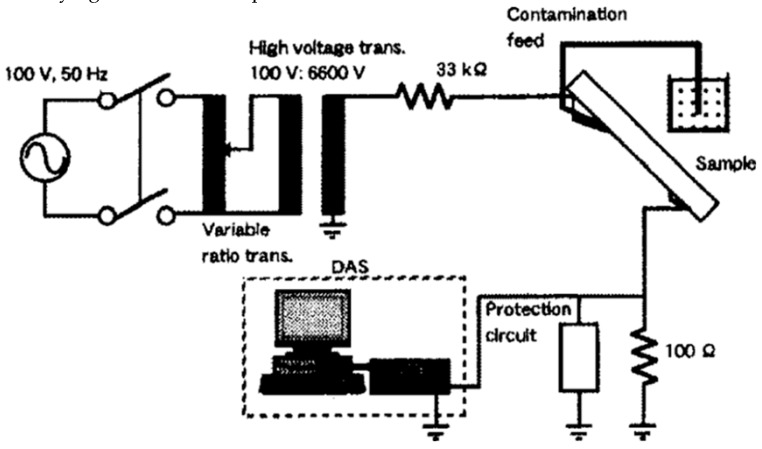
Schematic diagram of inclined plane test. The silicone rubber sample was fixed onto an inclined plane by two electrodes and contaminated liquid was dropped to the sample via filter paper.

The tracking and erosion test procedure for silicone rubber materials followed the inclined plane test (IPT) adopted Method 1—Application of constant tracking voltage in IEC-60587 [15], and the schematic diagram is shown in Figure 1 [16]. In the inclined plane test process, the voltage was increased to one of the test voltages list in Table 2 (i.e., 2.5, 3.5 or 4.5 kV) and applied to the standard size silicone rubber samples, and the conductive liquid was continuously and evenly dripped onto the sample surface with the purpose of generating intermittent arcing on the sample surface to simulate the ablation of the insulating material by the electric arcing. The schematic figure of the experiment setup is the same as that in [17].

All of the samples were cut into the standard size of 120∗50∗6 mm^3^ and tested in the experimental configuration sequence shown in Table 2. Each type of sample was tested 5 times under a certain experimental condition for 6 h. A type of sample would be considered to not have passed the test if one of the 5 samples showed overcurrent (exceeded 60 mA), penetrated hole or ignited during the test. The classifying method for samples is shown in Table 3.

### 2.3. Thermogravimetric Analysis

In this work, thermogravimetric analysis was used to validate the filler concentration of HTV silicone rubber materials. The thermo-gravimetric analyzer model used in this experiment is MDTC-EQ-M35-01. The temperature rose from 50 °C to 800 °C with a heating rate of 5 °C per minute.

### 2.4. LIBS Experimental Setup

**Figure 2:**
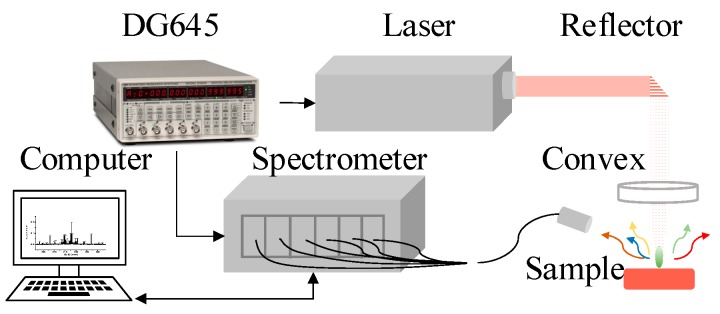
Schematic diagram of the LIBS setup. The LIBS setup includes a laser, a spectrometer, a digital delay generator, a computer and an optics system.

The LIBS system consisted of a laser, a spectrometer, a digital delay generator, an optical fiber, a computer and an optics system as shown in Figure 2. The laser model belongs to the Beamtech Nimma series, its output energy can reach up to 900 mJ when the laser wavelength is 1064 nm and its pulse duration is about 10 ns. The spectrometer has 6 channels corresponding to 6 different wave bands covering from 190 nm to 640 nm, and its sampling interval is approximately 0.01 nm. The synchronization between the laser shot and the spectrum collection process is realized by a DG645, which controls the delay between a laser shot and the beginning of spectrum collection. The LIBS test is conducted by focusing a laser onto the sample surface, ablating the material, and then the plasma is induced and emits the spectrum collected by the spectrometer via an optical fiber; eventually, the data are transferred to the computer for analysis. In this work, the time delay and the integration time of the spectrometer were set to 3 microseconds and 30 microseconds to archive an optimum signal-to-noise ratio, respectively. The energy of each laser pulse that actually arrived on the sample surface was measured by a laser-energy meter, and the average result was 64.5 mJ when the signal-to-noise ratio and signal-to-background ratio were both large enough to be analyzed.

## 3. Results

### 3.1. Tracking and Erosion Resistance of Samples

All types of samples were subjected to the inclined plane test, the details and a summary of the results are shown in Table 4 and Table 5. From the table, we can see that the silicone rubber material samples were classified as 1A0 when the ATH content was less than 0.1, classified as 1A2.5 when the ATH content was less than 0.8 and classified as 1A3.5 when the ATH content was less than 1.1. Only when the ATH content was greater than 1.1 were the samples classified as 1A4.5.

**Figure 3:**
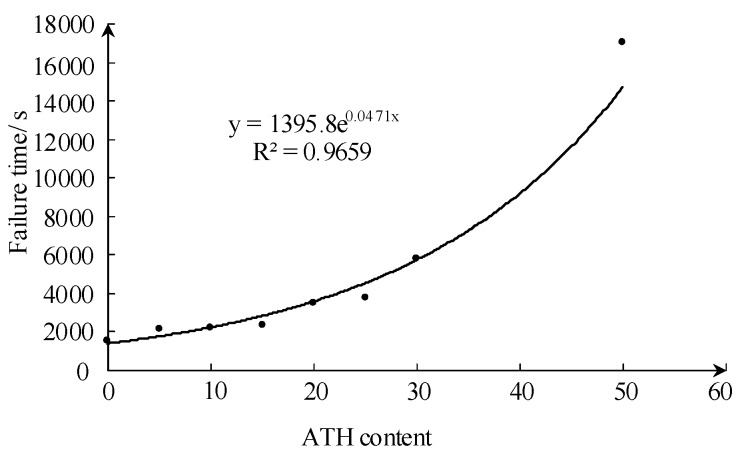
Failure time of IPT for different samples with different ATH contents under 3.5 kV test conditions.

The failure time of samples below Class 1A3.5 in the test with 3.5 kV is presented in Figure 3. As the ATH content increases, the tolerance time of the sample in the inclined plane test increases exponentially.

### 3.2. TGA Test

**Figure 4:**
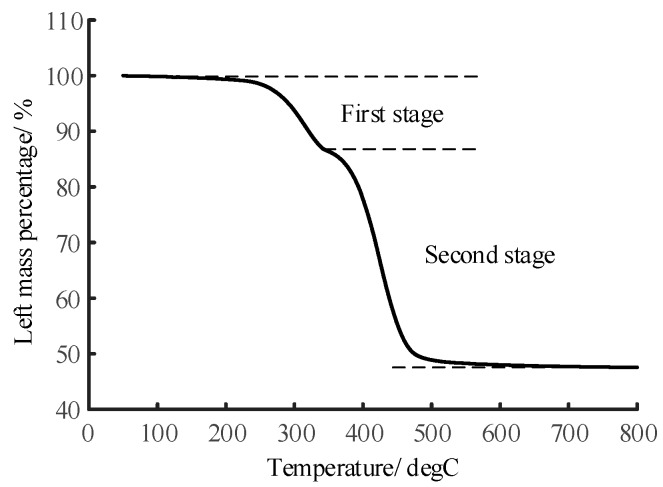
Thermogravimetric curve of silicone rubber (#23) whose ATH filler and silica filler mass fraction is 1.0 and 0.3, respectively, under a heating rate of 5 °C/K.

Thermo-Gravimetric Analysis (TGA) of 8 samples selected from the 27 samples was carried out under a heating rate of 5 °C/K. The 8 samples were selected because they have the most highly different ATH or silica content, covering the whole content range. It helps us to see clearly the relationship between the results of TGA and the results of LIBS measurement. Figure 4 shows how the residual mass fraction varies with constant temperature increase with time from 50 °C to 800 °C for the 8 samples.

The TG curves of silicone rubber samples with ATH filler added show 2 stages of reaction. The first stage is mainly about the decomposition of ATH from approximately 200 °C to 350 °C, and the second stage is about the decomposition of siloxane from approximately 350 °C to 600 °C. The thermo-gravimetric curves of samples with various ATH content and silica content in Table 4 have similar behaviors because the main ingredients are the same. The ATH content can be calculated based on the mass loss corresponding to the first stage, and the resin content is proportional to the mass loss in the second stage.

### 3.3. Emission Spectra

**Figure 5:**
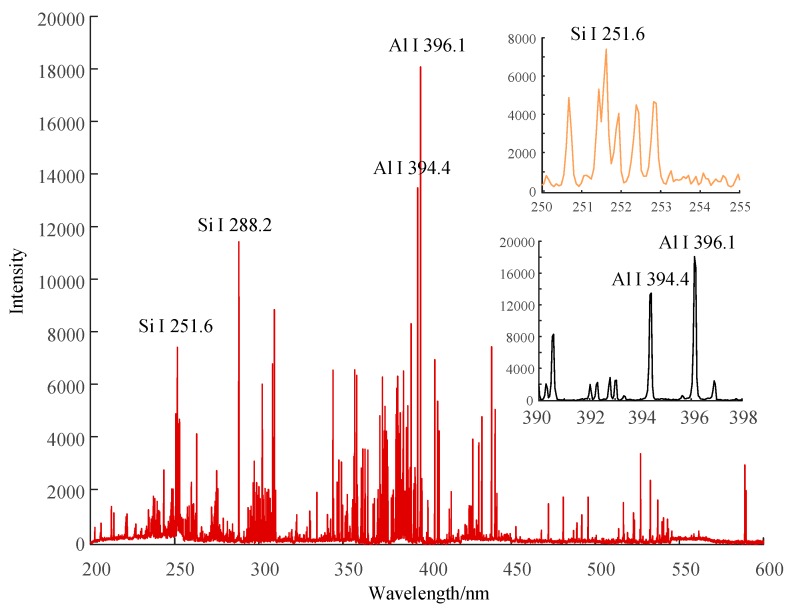
Emission spectrum of the new HTV silicone rubber for the first shot, while the mass fraction of its ATH and silica filler is 1.0 and 0.3, respectively.

A standard spectrum of HTV silicone rubber materials containing ATH and silica is shown in Figure 5. The peaks of the spectrum can be identified based on the NIST database. We can see that the intensity of Si and Al spectral lines is strong. Apparently, the Al element mainly exists in ATH filler, and the Si element exists in both silica filler and siloxane. There always exists the C element in the siloxane corresponding to the C I 247.9 nm in the spectrum. The Fe element is always present in the form of oxide in silicone rubber materials, and it has plenty of spectral lines.

**Figure 6:**
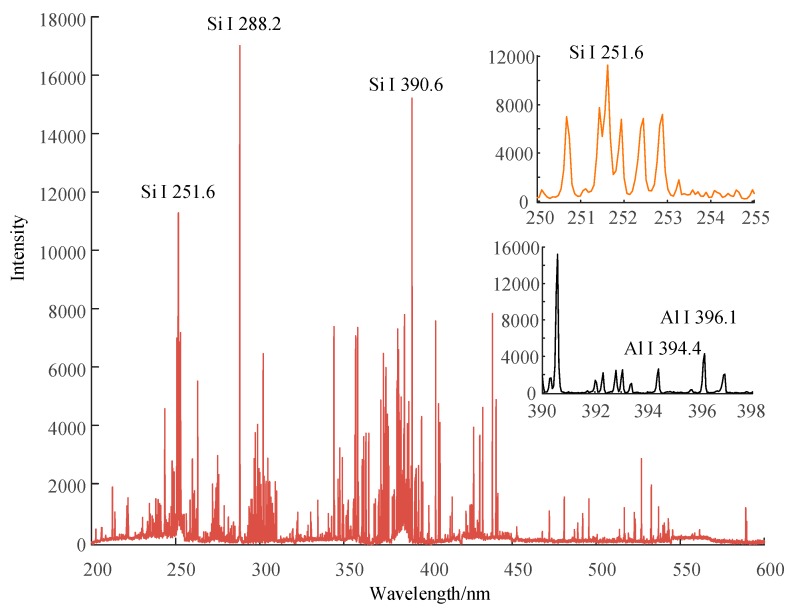
Emission spectrum of new HTV silicone rubber with no additional ATH and silica filler.

**Figure 7:**
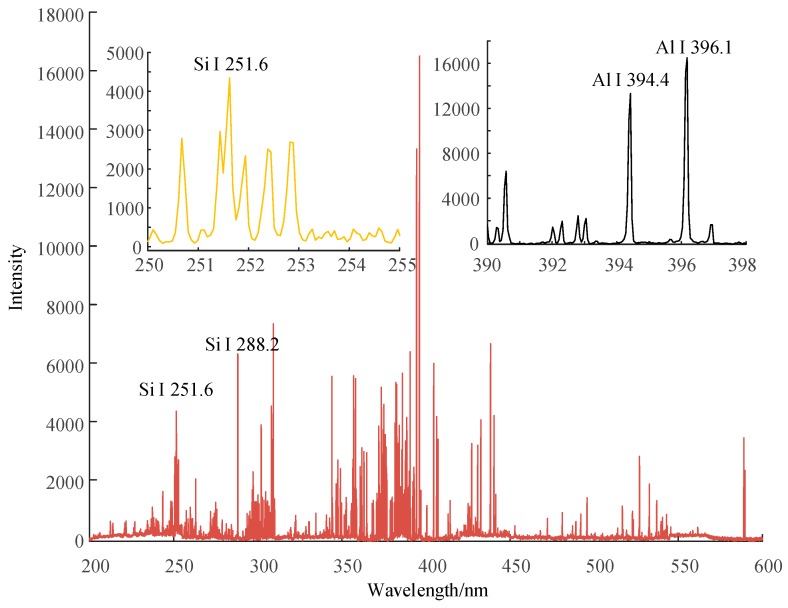
Emission spectrum of new silicone rubber with no silica filler, while the mass fraction of ATH is 1.5.

**Figure 8:**
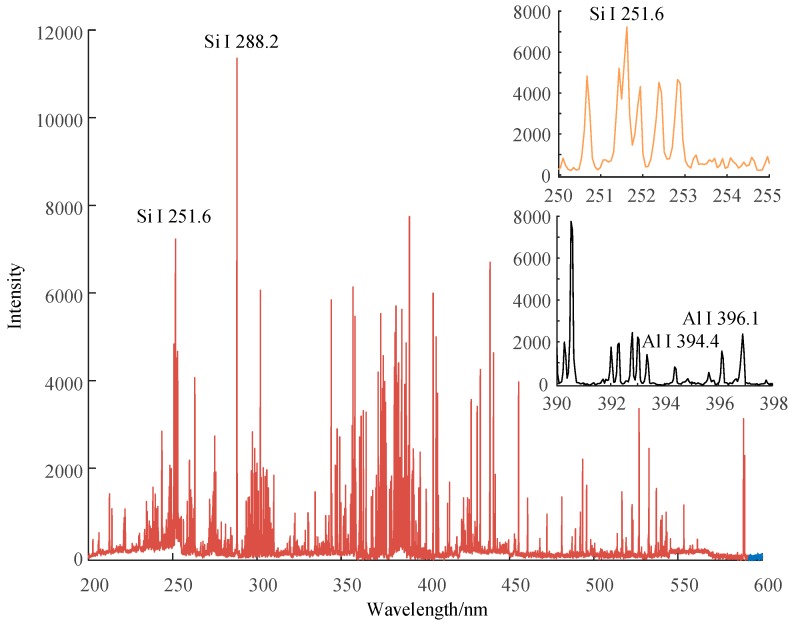
Emission spectrum of new silicone rubber with no ATH filler, while the mass fraction of silica filler is 0.3.

Figure 6, Figure 7 and Figure 8 show the emission spectrum of 3 types of new silicone rubber while the mass fraction of ATH is 0, 1.5, and 0, the mass fraction of silica filler is 0, 0, and 0.3, respectively. It can be seen that the number of spectral lines for samples without filler was less than that for samples with added silica filler, even though the filler did not contain any new element.

## 4. Discussion

### 4.1. Thermo-Gravimetric Analysis

**Figure 9:**
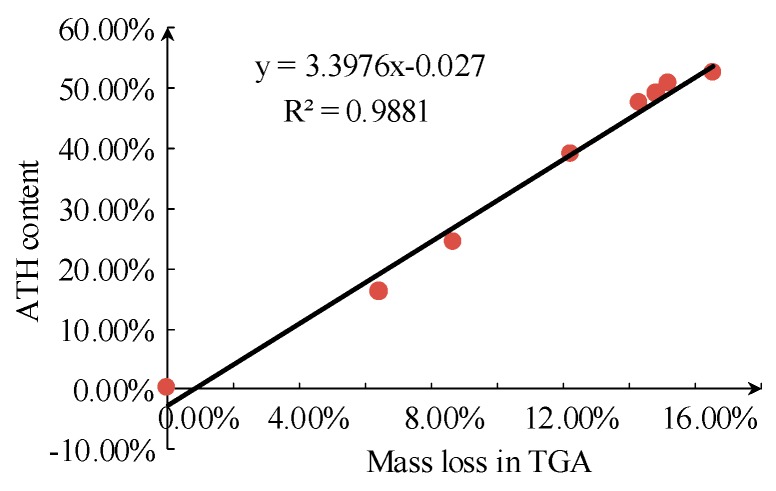
Relationship between actual ATH content and thermal mass loss in the first stage of thermogravimetric analysis.

**Figure 10:**
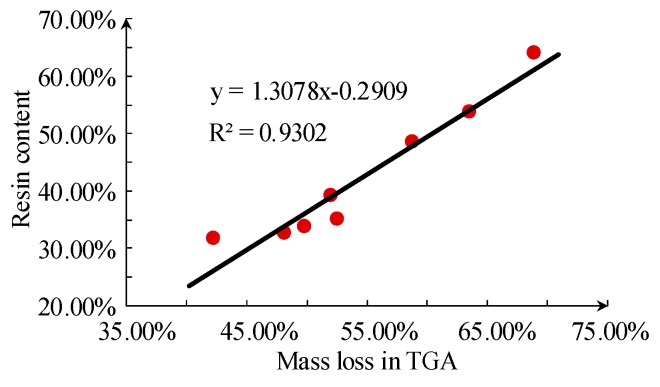
Relationship between base resin content and thermal mass loss in the second stage of thermogravimetric analysis.

By deriving the TG curve in Figure 3, the position of the turning point can be calculated, and the corresponding weight loss can be obtained. The weight loss at the position of the turning point corresponds to the total weight loss during the first stage, relating to the dehydration process of ATH filler. The second stage of TG is mainly regarding the decomposition of siloxane, and the corresponding weight loss is equal to the final weight loss minus the weight loss at the turning point. The results are shown in Figure 9 and Figure 10.

It can be seen that there is a close relationship between weight loss during the TG process and the ATH filler content of samples. The linear correlation coefficient between resin content and the corresponding weight loss is slightly lower, mainly because of the decomposition of other substances in that corresponding temperature range.

### 4.2. Emission Spectral Line

The tracking and erosion resistance performance of silicone rubber material is affected not only by filler content and resin content but also by other factors. This part will focus on the relationship between the main elements, especially Si, Al, C and O, and the corresponding spectral lines’ intensity. Our purpose is to find the relationships between the spectrum and the parameters of silicone rubber.

**Figure 11:**
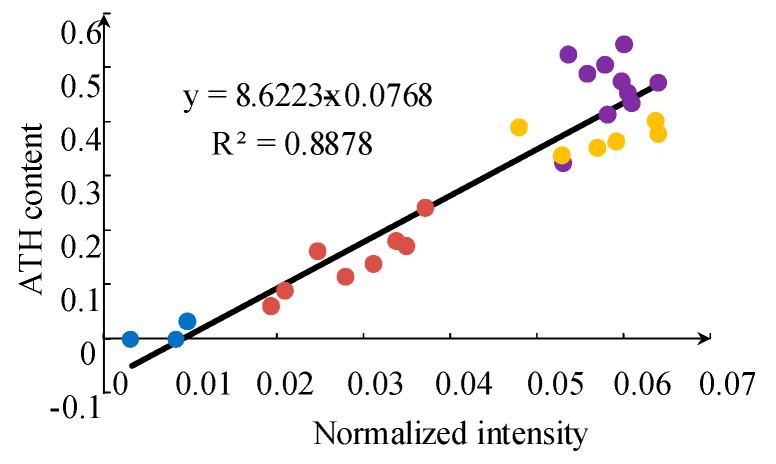
Normalized Al atomic line (Al I 308.2 nm) average intensity of samples with different ATH content; blue, red, yellow and violet points correspond to the silicone rubber samples classified as 1A0, 1A2.5, 1A3.5 and 1A4.5, respectively.

From the results of IPT, it is obvious that the ATH content has a decisive influence on the tracking and erosion resistance performance. It can be seen from Figure 11 that there is a positive correlation between the intensity of Al I 308.2 nm and ATH filler content as a whole. Notice that, when the ATH content reaches approximately 30%, the spectral intensity begins to saturate because of the self-absorption effect. The data points with the same tracking and erosion resistance performance are marked with the same color in Figure 11. It can be seen clearly from the figure that a higher tracking and erosion resistance grade corresponds to higher ATH content for the samples. A similar relationship exists between the tracking and erosion resistance grade and line intensity for Al I 308.2 nm. Samples under Class 1A3.5 can be easily distinguished by spectral data, while samples for 1A3.5 and 1A4.5 were difficult to distinguish when the corresponding ATH content was high.

**Figure 12:**
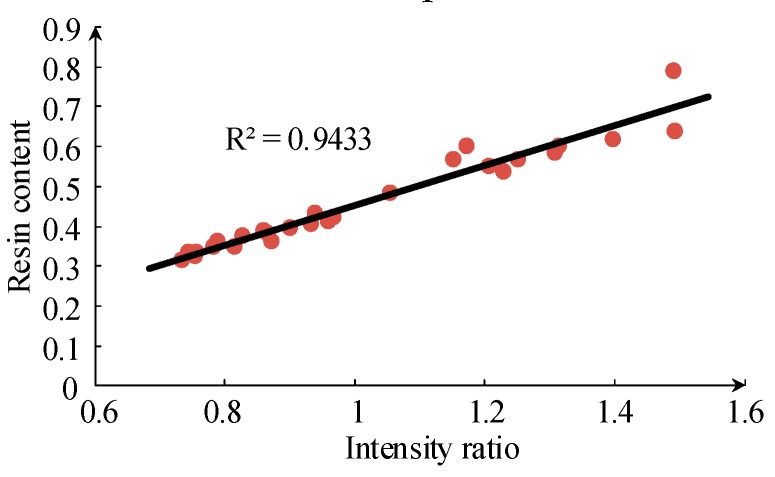
Intensity ratio of Si I 566.5 nm to C II 514.3 nm for samples with different resin content.

It is difficult to characterize the resin content of silicone rubber by spectrum data since both siloxane and silica filler contain Si. However, there is no C element, only Si and O elements, in silica filler, and the ratio of the Si and C spectral line intensity is considered to be used as the basis for the determination of the ablated substance. Figure 12 shows a good linear relationship between the atomic and ionic spectral line intensity ratio of Si to C and the resin content of the ablated silicone rubber material. Figure 12 shows a better result compared with TGA in Figure 10.

**Figure 13:**
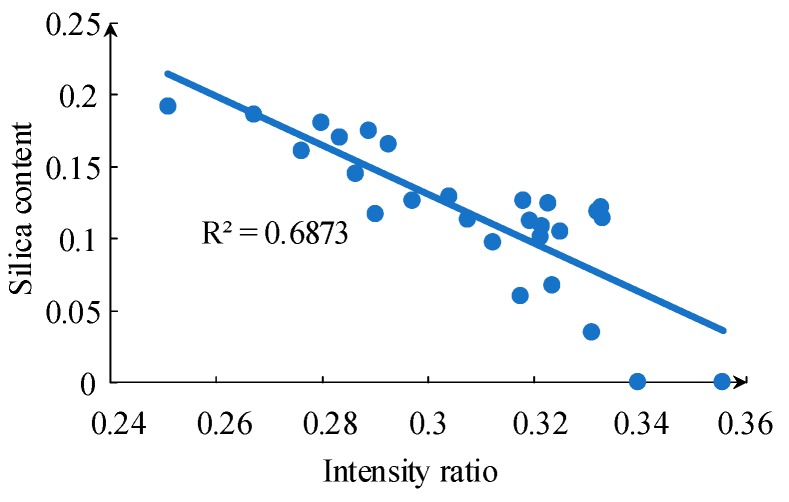
Intensity ratio of Si I 300.8 nm to O II 440.5 nm for samples with different silica filler content.

Silica filler can improve the mechanical behavior and thermal conductivity of silicone rubber. When more silica filler is added to the silicone rubber material, the oxygen content will obviously increase, and the ratio of Si element content to O element content will drop, as shown in Figure 13. However, Figure 13 shows a poor correlation between the spectrum and the silica content. This may be explained by the fact that Si and O elements exist in both siloxane and silica filler in silicone rubber materials, making it difficult to fully characterize the silica content with the spectral intensity ratio of Si and O elements.

**Figure 14:**
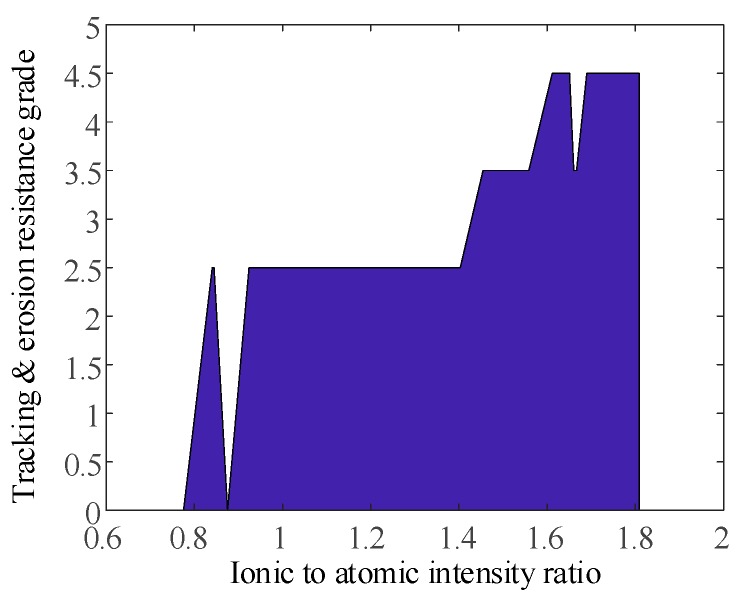
Relationship between the intensity ratio of Mg II 309.7 nm to Mg I 316.8 nm and the tracking and erosion resistance grade.

**Figure 15:**
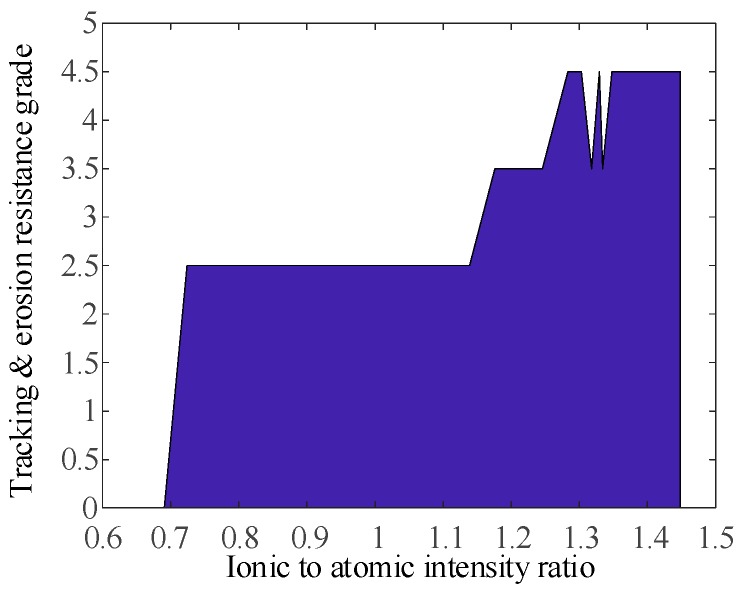
Relationship between the intensity ratio of Mg II 309.7 nm to Mg I 361.3 nm and the tracking and erosion resistance grade.

Moreover, we found that some spectral line intensity ratios of the Mg element can well characterize the tracking and erosion resistance performance of the samples, as shown in Figure 14 and Figure 15. The level is stepped up when the intensity ratio is gradually increased, except for several single points.

Through the analysis of the spectral data, it can be concluded that, though there always exist some single lines or line combinations to characterize the samples, the spectral line selection process is too complex to be used as a general method for silicone rubber materials. To make full use of the spectral data and simplify the spectral line selection process, the Principal component analysis (PCA) method was used in the following work.

### 4.3. Principal Component Analysis of LIBS Spectra

Principal component analysis is a data dimensionality reduction algorithm that transforms the original dataset onto a hyperplane. The standard orthonormal vectors of the hyperplane coordinate system are sorted according to the data variance projecting from the original space to the new space base vectors. The components with larger variances represent more features of the dataset. From the perspective of variance, usually most of the information in the data set can be characterized by the first few components.

PCA of the spectral dataset showed that the variances of the three largest components accounted for approximately 92% of the total variance, and the four largest components accounted for approximately 95%. An original spectrum contains approximately 10 thousand intensity data points for different wavelengths, while several components can be extracted to express most of the information via the PCA method, greatly simplifying the complexity of the subsequent computational model.

**Figure 16:**
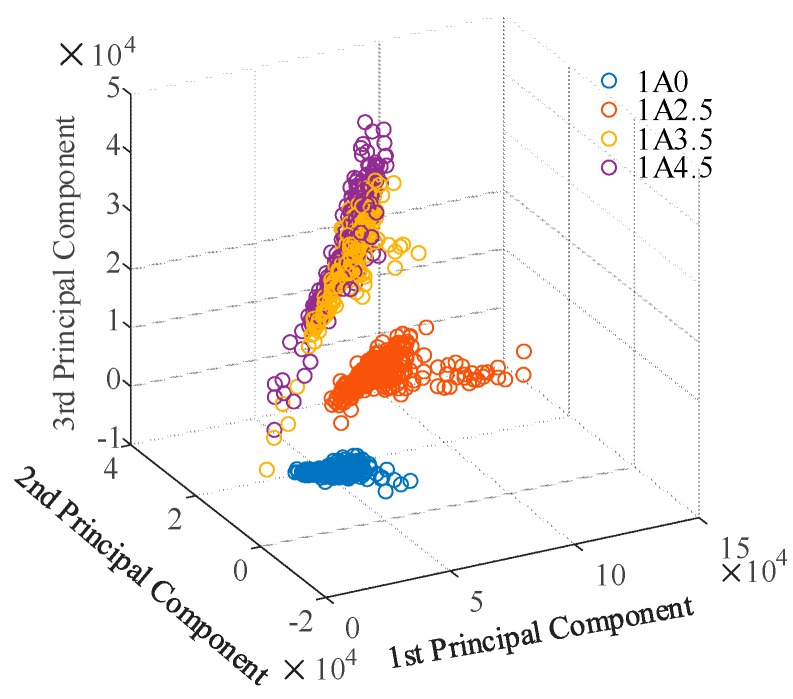
Distribution of spectral sets of samples for different tracking and erosion resistance performance in the new coordinate space.

Figure 16 shows the distribution of spectral data for four different-level samples in the new coordinate space using the three largest components. Data points corresponding to the same grade aggregate together, and point clouds of Class 1A0 and 1A2.5 were far away from other point clouds, while the point clouds of 1A3.5 and 1A4.5 were overlapping. This result indicates that it is easy to distinguish samples below grade 1A3.5 via a LIBS spectrum, but there may be problems for samples above 1A2.5. For this reason, a neural network algorithm was chosen to study the relationship between spectral data and the corresponding tracking and erosion resistance classification.

### 4.4. Neural Network Algorithm

**Figure 17:**
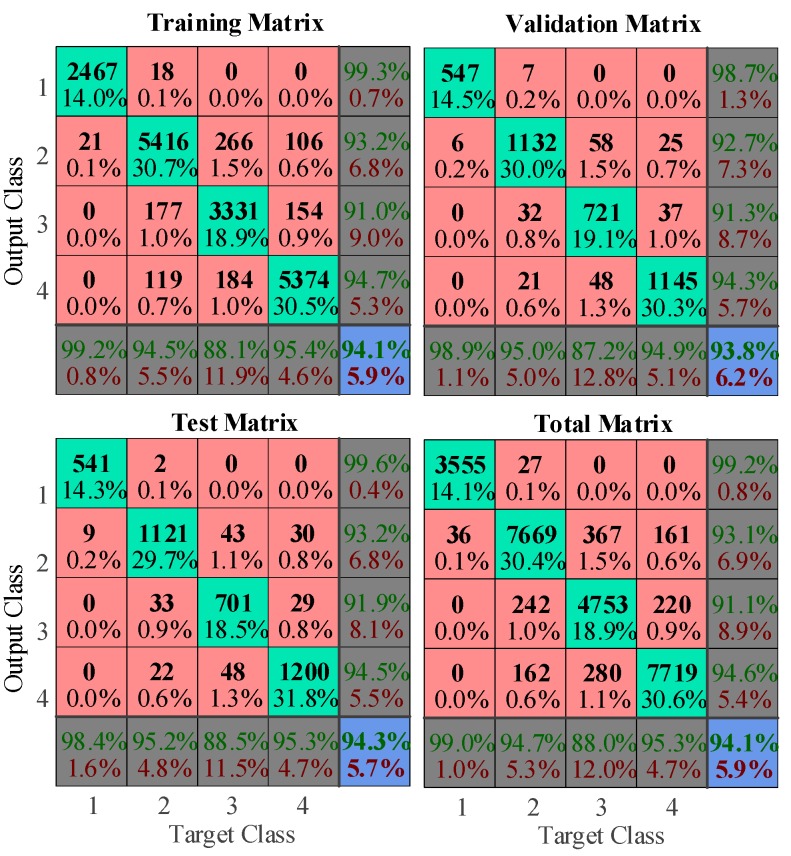
Confusion matrix of the neural network algorithm using the MATLAB neural net pattern recognition toolbox.

In this part, the neural network algorithm was used to classify the tracking and erosion resistance level of silicone rubber samples via LIBS spectral data. Spectral data was first processed by the PCA algorithm, and then the 8 largest principal components were selected as the input of the network, with the corresponding level as the output. 75% of the total spectral data was selected as the training set of the neural network, 15% was the validation set, and the remainder was left for the test set. The results of training are shown in Figure 17.

Because it is convenient for LIBS to make multiple measurements at the same point, we can calculate all the spectral data of samples to get the corresponding tracking and erosion resistance, and then the probability for each level of all the samples can be obtained. The tracking and erosion resistance with the highest probability was treated as the corresponding level of samples.

There were 27 types of samples in total, including 3 samples classified as Class 1A0, 9 samples classified as 1A2.5, 6 samples classified as 1A3.5, and 9 samples classified as 1A4.5; finally, all of the samples were recognized correctly, and some of the results are shown in Table 6 through Table 9. It can be seen from Table 6 and Table 7 that silicone rubber material classified below Class 1A3.5 has a reliably nice recognition result. The calculated probability for the corresponding level was greater than 90%. This is consistent with the discussion of PCA in the previous section. However, it is slightly dangerous for the recognition of samples classified as 1A3.5 in the method since one of the samples was nearly recognized as 1A2.5, as shown in the second column in Table 8. Our model thought that this sample had a 57.6% probability of being classified as 1A3.5 and a 31.3% probability of being classified as Class 1A2.5. This can be explained by the fact that this sample contains 0.8 of ATH filler, while a sample with 0.75 of ATH filler cannot pass the test with 3.5 kV. Comparing the second sample in Table 8 and the first sample in Table 9, it can be seen that there is a certain effect on the probability of grading because of their relatively close composition. This is also consistent with Figure 16.

From the above discussion, it can be seen that, although some samples have a relatively low probability for the correct tracking and erosion resistance classification, the final recognition is still correct when the level with highest probability is selected. All the samples were recognized correctly using hundreds of spectra for each sample.

## 5. Conclusions

In this paper, the tracking and erosion resistance of silicone rubber material was analyzed using the LIBS technique. Different samples were prepared to study the range of filler content corresponding to different tracking and erosion resistance levels. The results of filler content using TGA and LIBS were compared. Conventional methods of LIBS were used to study the tracking and erosion resistance of samples. Some specific single lines or line combinations can be used to characterize the performance, requiring tedious calculations and screening. PCA was used to extract the principal components of spectral data to avoid tedious line selection work and to use the total information of full spectra as much as possible. The 8 largest principal components were used as the input of the neural network, while the corresponding output was the tracking and erosion resistance classification. The tracking and erosion resistances of all spectra of each sample were calculated, and the classification with the highest probability for each sample was obtained. In summary, LIBS can effectively measure the tracking and erosion resistance of silicone rubber material using PCA and neural network algorithms.

## Figures and Tables

**Table 1:** Concentrations of 27 samples. The numerical value represents mass fraction taking resin content as a reference.

Index	Resin	ATH	Silica	Other Assistance
1	1	0.05	0.3	0.264
2	1	0.1	0.3	0.264
3	1	0.15	0.3	0.264
4	1	0.2	0.3	0.264
5	1	0.25	0.3	0.264
6	1	0.3	0.2	0.264
7	1	0.3	0.1	0.264
8	1	1.1	0.3	0.264
9	1	1.2	0.3	0.264
10	1	1.3	0.3	0.264
11	1	1.4	0.3	0.264
12	1	0.75	0.3	0.264
13	1	1.5	0	0.264
14	1	0.8	0.3	0.264
15	1	0.85	0.3	0.264
16	1	0.9	0.3	0.264
17	1	0.95	0.3	0.264
18	1	1.05	0.3	0.264
19	1	0	0	0.264
20	1	0	0.3	0.264
21	1	0.3	0.3	0.264
22	1	0.5	0.3	0.264
23	1	1	0.3	0.264
24	1	1.5	0.3	0.264
25	1	1.5	0.1	0.264
26	1	1.5	0.2	0.264
27	1	1.5	0.4	0.264

**Table 2:** Configuration of the inclined plane test for different testing levels.

Test Voltage (kV)	Preferred Test Voltage (kV)	Contaminant Flow Rate mL/min	Series Resistor, Resistance (kΩ)
2.0–2.75	2.5	0.15	10
3.0–3.75	3.5	0.3	22
4.0–4.75	4.5	0.6	33

**Table 3:** Grading criterion for the inclined plane test.

Classification Result	Test Voltage—2.5 kV	Test Voltage—3.5 kV	Test Voltage—4.5 kV
1A0	fail	-	-
1A2.5	pass	fail	-
1A3.5	-	pass	fail
1A4.5	-	-	pass

**Table 4:** The results of the inclined plane test for all the samples.

Index	ATH	Silica	Tracking and Erosion Resistance Classification
1	0.05	0.3	1A0
2	0.1	0.3	1A2.5
3	0.15	0.3	1A2.5
4	0.2	0.3	1A2.5
5	0.25	0.3	1A2.5
6	0.3	0.2	1A2.5
7	0.3	0.1	1A2.5
8	1.1	0.3	1A4.5
9	1.2	0.3	1A4.5
10	1.3	0.3	1A4.5
11	1.4	0.3	1A4.5
12	0.75	0.3	1A2.5
13	1.5	0	1A4.5
14	0.8	0.3	1A3.5
15	0.85	0.3	1A3.5
16	0.9	0.3	1A3.5
17	0.95	0.3	1A3.5
18	1.05	0.3	1A3.5
19	0	0	1A0
20	0	0.3	1A0
21	0.3	0.3	1A2.5
22	0.5	0.3	1A2.5
23	1	0.3	1A3.5
24	1.5	0.3	1A4.5
25	1.5	0.1	1A4.5
26	1.5	0.2	1A4.5
27	1.5	0.4	1A4.5

**Table 5:** Summary of the tracking and erosion resistance classification for all samples and the corresponding test condition.

ATH Content Range	Tracking and Erosion Resistance Grade
0–5	1A0
10–75	1A2.5
80–105	1A3.5
110–150	1A4.5

**Table 6:** Recognition results of the tracking and erosion resistance of samples below 1A0 using the LIBS technique based on the neural network algorithm.

Classification	1	2	3
1A0	96.3%	100.0%	99.7%
1A2.5	3.7%	0.0%	0.3%
1A3.5	0.0%	0.0%	0.0%
1A4.5	0.0%	0.0%	0.0%

**Table 7:** Recognition results of the tracking and erosion resistance of samples classified as 1A2.5 using the LIBS technique based on the neural network algorithm.

Classification	1	2	3	4
1A0	2.3%	0.7%	0.0%	0.0%
1A2.5	97.7%	99.3%	99.9%	99.6%
1A3.5	0.0%	0.0%	0.0%	0.1%
1A4.5	0.0%	0.0%	0.1%	0.3%

**Table 8:** Recognition results of the tracking and erosion resistance of samples classified as 1A3.5 using the LIBS technique based on the neural network algorithm.

Classification	1	2	3	4
1A0	0.0%	0.0%	0.0%	0.0%
1A2.5	31.3%	6.8%	0.2%	0.3%
1A3.5	57.6%	75.7%	99.3%	99.0%
1A4.5	11.3%	17.6%	0.4%	0.7%

**Table 9:** Recognition results of the tracking and erosion resistance of samples classified as 1A 4.5 using the LIBS technique based on the neural network algorithm.

Classification	1	2	3	4
1A0	0.0%	0.0%	0.0%	0.0%
1A2.5	4.3%	0.7%	3.1%	1.8%
1A3.5	6.9%	1.4%	2.2%	0.6%
1A4.5	88.8%	97.9%	94.8%	97.9%
